# Impact of Model Shape Mismatch on Reconstruction Quality in Electrical Impedance Tomography

**DOI:** 10.1109/TMI.2012.2200904

**Published:** 2012-05-22

**Authors:** Bartłomiej Grychtol, William R. B. Lionheart, Marc Bodenstein, Gerhard K. Wolf, Andy Adler

**Affiliations:** 1 Department of Medical Physics in RadiologyGerman Cancer Research Centre (DKFZ) Heidelberg 69120 Germany; 2 School of MathematicsUniversity of Manchester Manchester M13 9PL England; 3 Department of AnesthesiologyJohannes Gutenberg-University Mainz Mainz Germany; 4 Division of Critical Care MedicineDepartment of AnesthesiologyChildren's Hospital BostonHarvard Medical School Boston MA 02115 USA; 5 Systems and Computer EngineeringCarleton University Ottawa ON K1S 5B6 Canada

**Keywords:** Electrical impedance tomography (EIT), mechanical ventilation, model, reconstruction, shape

## Abstract

Electrical impedance tomography (EIT) is a low-cost, noninvasive and radiation free medical imaging modality for monitoring ventilation distribution in the lung. Although such information could be invaluable in preventing ventilator-induced lung injury in mechanically ventilated patients, clinical application of EIT is hindered by difficulties in interpreting the resulting images. One source of this difficulty is the frequent use of simple shapes which do not correspond to the anatomy to reconstruct EIT images. The mismatch between the true body shape and the one used for reconstruction is known to introduce errors, which to date have not been properly characterized. In the present study we, therefore, seek to 1) characterize and quantify the errors resulting from a reconstruction shape mismatch for a number of popular EIT reconstruction algorithms and 2) develop recommendations on the tolerated amount of mismatch for each algorithm. Using real and simulated data, we analyze the performance of four EIT reconstruction algorithms under different degrees of shape mismatch. Results suggest that while slight shape mismatch is well tolerated by all algorithms, using a circular shape severely degrades their performance.

## I. Introduction

Electrical impedance tomography (EIT) is a promising medical imaging modality for monitoring ventilation distribution in the lung. In thoracic EIT, imperceptible current injection and voltage measurement through surface electrodes around the thorax are used to reconstruct a conductivity map across a transverse slice of the body. EIT is low-cost, noninvasive, radiation free, and available at the bedside. One of the most promising applications of EIT is for monitoring and/or guiding mechanical ventilation therapy. The ability of EIT to measure regional distribution of ventilation has been validated against single photon emission computed tomography (SPECT) [1], X-ray computed tomography (CT) [2], [3], and positron emission tomography (PET) [4]. No other currently available technology can provide real-time long term monitoring of the regional functional state of the lungs. Although such information could be invaluable in preventing ventilator-induced lung injury (VILI), clinical application of EIT is hindered by difficulties in interpreting the resulting images.

Such difficulties are often a result of errors in the forward modeling of the electrical fields, a necessary step in reconstructing the conductivity distribution. In particular, no 2-D model can fit EIT data obtained from a 3-D domain (body) [5] and, even when a 3-D model of a domain is used, it is generally impossible to accurately fit data from an isotropic conductivity distribution if the boundary shape is wrong [6].

Because in clinical practice the boundary shape is generally unknown and changes with breathing and posture, the problem is often reduced to reconstructing the changes rather than absolute conductivity, which is less sensitive to shape mismatch and easier to solve. A circular shape has traditionally been used to represent a cross section of the subject's body [7]. This lack of correspondence to the anatomy imposes several limitations on the analysis of EIT images. Because expected organ shape and position on circular images is unknown, it is difficult to distinguish some artifacts from correct images. Images of different patients cannot be directly compared. Moreover, the mismatch between the true body shape and the shape used for reconstruction is known to produce image errors [8], [6], which to date have not been properly characterized.

In a preliminary study of one reconstruction algorithm [9], we showed that using the correct body shape obtained from a CT scan produces reconstructions qualitatively superior to those produced with a circular shape. However, for practical reasons, EIT reconstruction cannot depend on the availability of a CT scan of each individual subject. Patient shape could instead be obtained by means of, for example, wearable sensors or through optical 3-D surface reconstruction (from images obtained with a multi-camera system). However, we believe that developing a set of predefined shapes to choose from for each patient based on easy to measure parameters (weight, height, etc.) is the most practical and least expensive approach. In order to develop such a set, a deeper understanding of the errors and tolerances of different EIT algorithms with respect to shape mismatch is required.

In the present study we, therefore, seek to 1) characterize and quantify the errors resulting from reconstruction shape mismatch for a number of popular EIT reconstruction algorithms and 2) develop recommendations on the tolerated amount of mismatch for each algorithm.

## II. Methods

Shortly, the external boundary shape of a human and a swine were obtained from sample CT images in the electrode plane. From each so obtained true shape a number of progressively more circular contours were derived. For each contour a 3-D finite element model (FEM) was built by extrusion along the long axis of the body. The models were used to reconstruct simulated and real data using four EIT reconstruction algorithms.

**Fig. 1.**
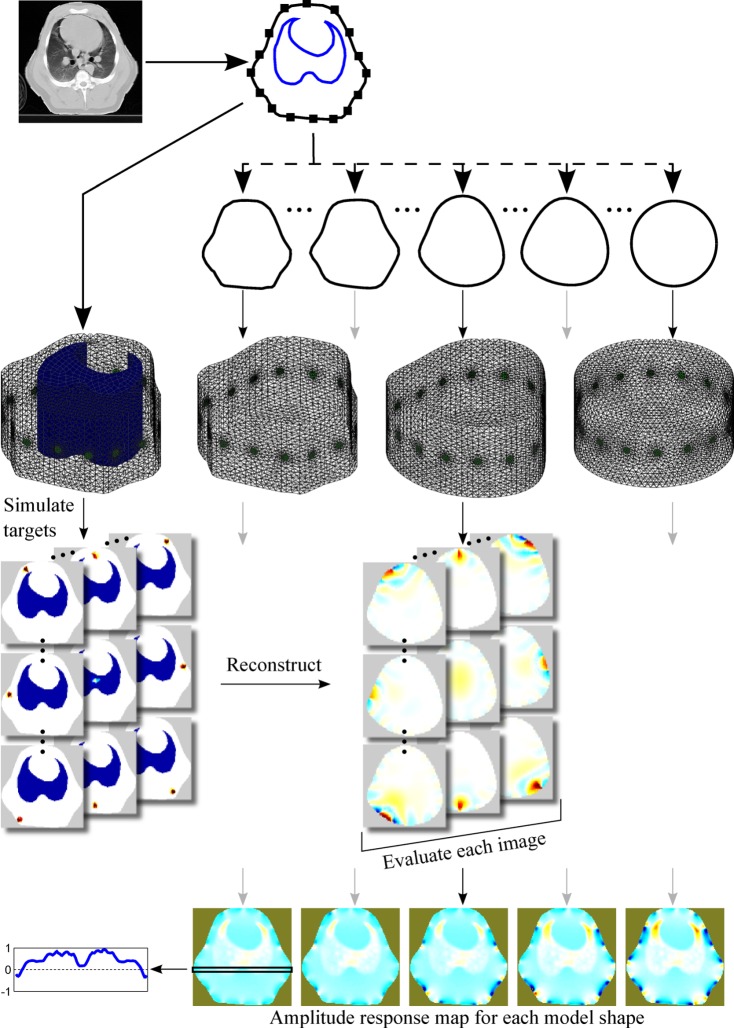
Overview of the methods. A CT slice in the electrode plane is segmented to obtain the boundary shape, the contour of the lungs and the electrode positions. An FEM with a lung contrast conforming to the CT slice is created and used to simulate a number of targets covering the whole body. Several homogeneous models with distorted shape are created and used for reconstruction. Based on the individual target reconstructions, a map representing performance metric as a function of position is constructed for each model shape.

The results were evaluated in terms of the performance measures agreed by a representative group of researchers and practitioners in the field [10]. The procedure is presented schematically in Fig. 1.

All calculations have been carried out with Matlab (Mathwarks, Natick, MA) using the EIDORS^1^^1^http://eidors3d.sourceforge.net/ toolbox [11], to which all relevant tools developed as part of this project have been contributed and were included in the latest release (3.5).

### A. Model Shape

To investigate the impact of mismatch between the actual body shape and that of the FEM used for EIT image reconstruction, a number of progressively more inaccurate shapes were obtained as follows. First, the true shape was obtained from a single CT image of the thorax at the electrode plane by manual delineation with a number of points (37 for the human

**Fig. 2.**
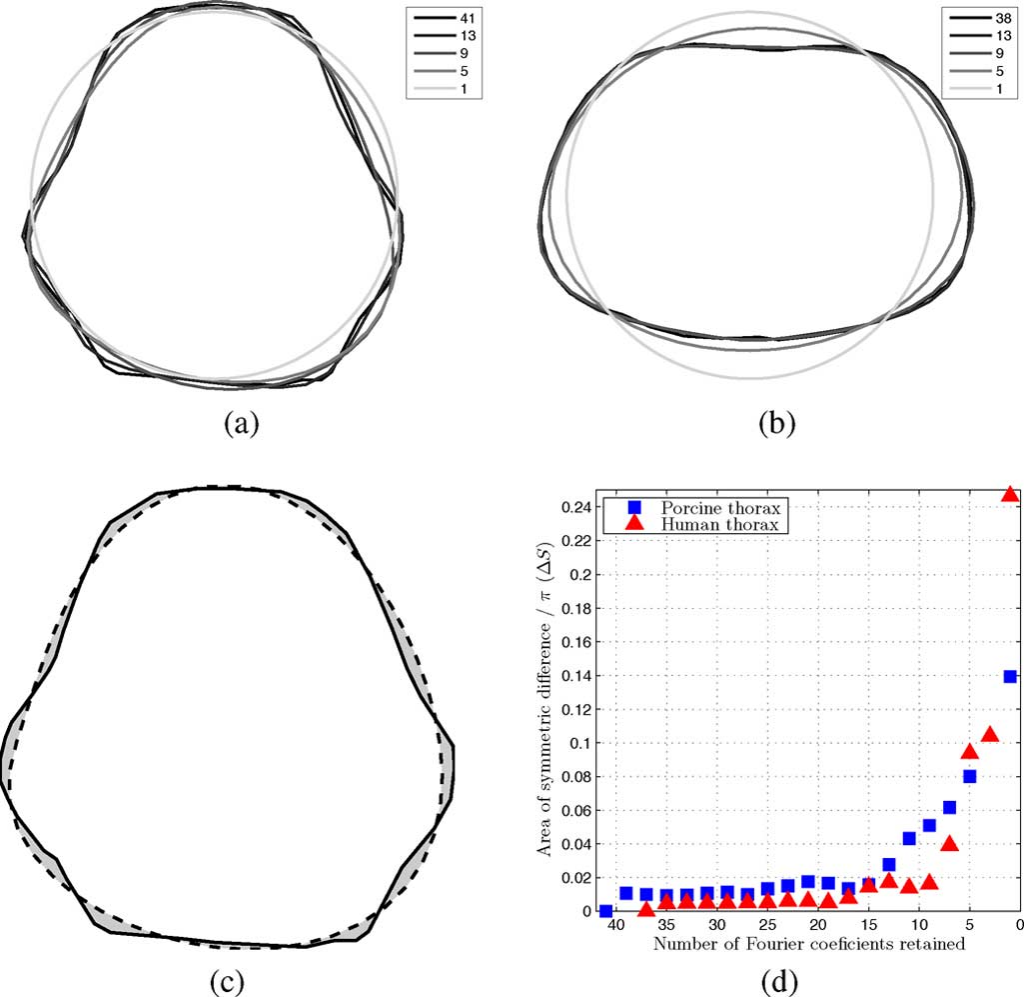
Representative swine (a) and human (b) thorax model shapes. Legends indicate the number of retained Fourier coefficients. (c) Area of symmetric difference between the original pig thorax shape and a smooth one. (d) Area of symmetric difference for both human and porcine model shapes as a function of the number of Fourier coefficients retained.

shape and 41 for the swine). The original pixel coordinates of the resulting points were rigidly transformed such that the entire shape fitted in a square with side 2 centered at the origin. All subsequents unit-less quantities are reported in this coordinate system. Second, the points were interpreted in the complex plane (with origin coinciding with the Cartesian coordinates just defined) and a parametric description of the shape was obtained by taking the discrete Fourier transform (DFT) of the resulting complex vector. Third, progressively smoother shapes were obtained by truncating the Fourier series, two components at a time, down to a length of five terms (at 3 the resulting shape was an almost perfect circle but off-center with respect to the other shapes). At each series length a new shape of 41–45 equidistant points, the exact number adjusted to allow the creation of a finite element model with Netgen [12] as described below, was obtained by padding the truncated Fourier series with zeros and taking the inverse DFT. All shapes were scaled to have the same area as the original $(\pi)$. The last shape was a circle with radius 1. Representative shapes for both animal and human geometries are presented in Fig. 2.

The mismatch $\Delta S$ between a smooth shape and the original was quantified as the area of symmetric difference (nonoverlapping area) between the two shapes (divided by $\pi$), as illustrated in Fig. 2(c). Fig. 2(d) presents $\Delta S$ as a function of the number of Fourier coefficients retained (assuming 1 for the circular model).

In order to create 3-D models from thus defined contours, we extended the EIDORS interface to Netgen [12] to support extrusion. For each shape we then created a 3-D model by extruding the 2-D outline to a height of 1. Sixteen circular electrodes were placed equidistantly around the perimeter of the model at a height of 0.5. The mesh was refined locally around the electrodes. Occasionally the locations of the electrodes and the outline points interacted in ways that prevented Netgen from successfully meshing the geometry. In such cases, the number of points describing a shape was increased, as mentioned earlier. Sample meshes are presented in Fig. 1.

### B. Reconstruction Algorithms

The reconstruction of conductivity values inside a body based on surface voltage measurements is a severely ill-posed nonlinear inverse problem. However, because of the large uncertainties about measurement noise, domain shape and electrode impedance present in clinical and experimental EIT data alike, various linearized approximations to solving difference data have proven useful. In difference EIT, a vector of conductivity change ${\bf x}={\mbi \sigma} - {\mbi \sigma}_{\rm r}$ between the current conductivity ${\mbi \sigma}$ and the reference ${\mbi \sigma}_r$ is reconstructed from measurements ${\bf y} = {\bf v} - {\bf v}_{\rm r}$ of the corresponding change in recorded voltage. Often, both differences are element-wise normalized (such that $y_i=(v_i-v_{{\rm r}i})/v_{{\rm r}i})$, as is also the case in the present study.

For sufficiently small changes, the relationship between ${\bf x}$ and ${\bf y}$ can be approximated by the linear relationship

$${\bf y} = {\bf J} {\bf x} + {\bf n} \eqno{\hbox{(1)}}$$

where ${\bf J}$ is the Jacobian or sensitivity matrix calculated for each element of the FEM as $J_{ij}=({\partial y_i})/({\partial x_j})$ and ${\bf n}$ represents the measurement noise (assumed to be uncorrelated white Gaussian). Because the number of conductivity elements is much greater than the number of measurements, ${\bf x}$ is longer than ${\bf y}$, and ${\bf J}$ is not square and therefore does not have an inverse. Instead, a linear reconstruction algorithm calculates an estimate of ${\bf x}$

$${\mathhat{\bf x}} = {\bf R} {\bf y} \eqno{\hbox{(2)}}$$

using a reconstruction matrix ${\bf R}$. Many algorithms to derive ${\bf R}$ have been proposed, four of which are used in this study: TSVD (truncated singular value decomposition [13], [14]), GREIT (Graz consensus Reconstruction algorithm for EIT [10]), and two variants of the one-step Gauss–Newton (GN) method.

In the TSVD algorithm, ${\bf R}$ is the truncated pseudoinverse

$${\bf J}^+_{\rm t} = {\bf V}{\bf D}^+_{\rm t}{\bf U}^* \eqno{\hbox{(3)}}$$

of ${\bf J}$, where ${\bf J} = {\bf U}{\bf D}{\bf V}^*$ is the singular value decomposition of ${\bf J}$ and ${\bf D}^+_{\rm t}$ is obtained as

$${\bf D}^+_{\rm t}[i,i] = \cases{{\bf D}[i,i]^{-1},& $ {\rm if}\ \Vert {\bf D} [i,i]\Vert \geq t $\cr 0, & $ {\rm otherwise}$.} \eqno{\hbox{(4)}}$$

As the threshold $t$ is increased less components of ${\bf D}$ are retained, which means that only the more significant singular values are used, thus increasing the amount of regularization.

The GREIT reconstruction matrix is calculated from simulated measurements ${\bf Y}$ and the corresponding desired solutions $\mathtilde{\bf X}$ as

$${\bf R} = \mathtilde{\bf X} {\bf Y} ({\bf J} {\bf \Sigma}_{\rm x} {\bf J}^{\rm T} + \lambda{\bf \Sigma}_{\rm n})^{-1} \eqno{\hbox{(5)}}$$

where $\mathtilde{\bf X} = ({1})/({n}) (\mathtilde{\bf x}^{(1)} \ldots \mathtilde{\bf x}^{(n)})$ and ${\bf Y} = ({1})/({n})({\bf y}^{(1)} \ldots {\bf y}^{(n)})$ are matrixes obtained by horizontal concatenation of $n$ desired solution or simulated measurement vectors, respectively, while ${\bf \Sigma}_{\rm n}$ and ${\bf \Sigma}_{\rm x}$ represent the noise and image covariance matrixes [10]. The trade-off between the different performance measures is embedded in the desired solutions while the hyperparameter $\lambda$ controls the amount of regularization. We extended the original implementation of the GREIT algorithm, previously only defined for cylinders, to arbitrary shapes [9].

For the GN algorithms, ${\bf R}$ can be expressed as

$${\bf R} = ({\bf J}^{\rm T} {\bf J} + \lambda {\bf P})^{-1} {\bf J}^{\rm T} \eqno{\hbox{(6)}}$$

where ${\bf P}$ is a regularization prior matrix and $\lambda$ is again a hyperparameter controlling the amount of regularization. We test the NOSER [15] and discrete Laplace filter [16] priors. We use the normalized difference imaging approach, whereby conductivity differences are not reconstructed as absolute values but as unit-less ratios to the reference background conductivity, defined as an expiration or target-less simulation measurement for clinical and simulation data, respectively.

All tested algorithms employ the dual model approach whereby the Jacobian ${\bf J}$ is calculated on a 3-D forward model obtained as detailed above, but values are only reconstructed on a 2-D rectangular grid in the electrode plane. After [10], we adjusted the hyperparameter value for each model and algorithm such as to achieve noise amplification (as defined by the Noise Figure parameter in [17]) of 0.5 in the center of the image. This method of choosing the regularization parameter is configuration-independent and has been shown to consistently produce good reconstructions [18].

### C. Evaluation Criteria

For each shape, each reconstruction algorithm was evaluated using the performance figures of merit defined in [10]. Briefly, these are: amplitude response (AR), resolution (RES), shape deformation (SD), position error (PE), ringing (RNG), and position error (PE). Each figure of merit is measured

**Fig. 3.**
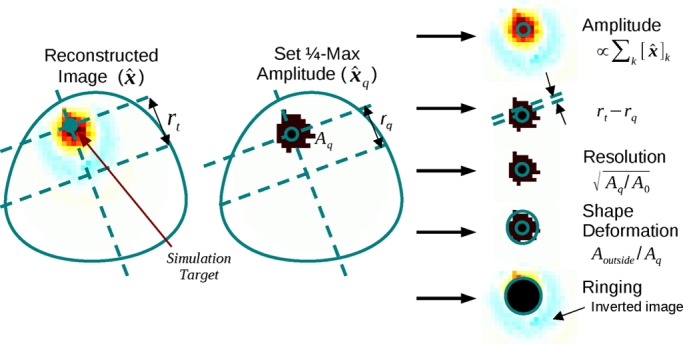
Algorithm evaluation criteria calculated by comparing the desired and actual reconstructed images. Adapted from [10].

empirically on a reconstructed image of a small simulated target, as illustrated in Fig. 3.

To evaluate the spatial variability of each algorithm with respect to the performance measures, calculations were carried out for a large set of regularly spaced small targets in the electrode plane. Thus, for each shape and figure of merit, the performance of the algorithm is represented as an image with each pixel corresponding to one simulated target and its value reflecting the respective figure of merit obtained by reconstructing that single simulated target. The mean of values within each such image and the ratio of standard deviation to the mean are analyzed as a function of the shape deformation $\Delta S$.

### D. Simulation

Simulated measurements were obtained through the FEM method using a mesh with the true thorax shape (obtained as described above) and a conductivity contrast in the lung region segmented from the corresponding CT image. The lung to other tissue conductivity ratio was 0.1875 (as the average of the expiration and inspiration values assumed in [8] and in agreement with the ranges observed by Gabriel et al. [19] for 100 kHz current frequency). This simulation setup represents well the practical use of EIT where measurements obtained on a heterogeneous body are reconstructed using a homogeneous model. The meshes of the human and pig chest contained $31\times 10^3$ and $33\times 10^3$ first-order tetrahedral elements, respectively.

### E. Data

The animal data used in this study were obtained at the University of Mainz, Germany, under appropriate ethical approval (license no. 1.5 177-07/041-75, Landesuntersuchungsamt Rheinland-Pfalz, 56028 Koblenz, Germany). CT data were acquired during a period of apnea in a healthy 23 kg swine. EIT data were recorded during

**Fig. 4.**
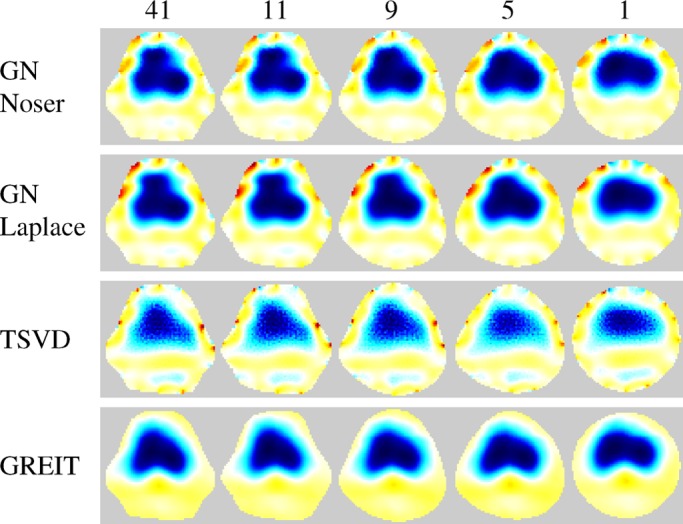
Reconstructions on various model shapes of data from a healthy pig. Images show difference between inspiration and expiration in one breath cycle. The number of Fourier coefficients used to define each shape is indicated at the top.

conventional mechanical ventilation in the same animal.

The human CT used in this study originates from a diagnostic scan of a male volunteer (54 year old, BMI 25.4, healthy lung and heart) taken to investigate a nonthoracic condition and donated by the subject to the EIDORS project for scientific purposes.

## III. Results

### A. Animal Experiment Data

Sample reconstructed images of animal data using all four algorithms are presented in Fig. 4. For all algorithms, the more circular the model shape is, the more distorted the lung shape appears. Features along the longer vertical axis are pushed together and lost, particularly at the ventral side. Qualitatively, images reconstructed with the GREIT algorithm

**Fig. 5.**
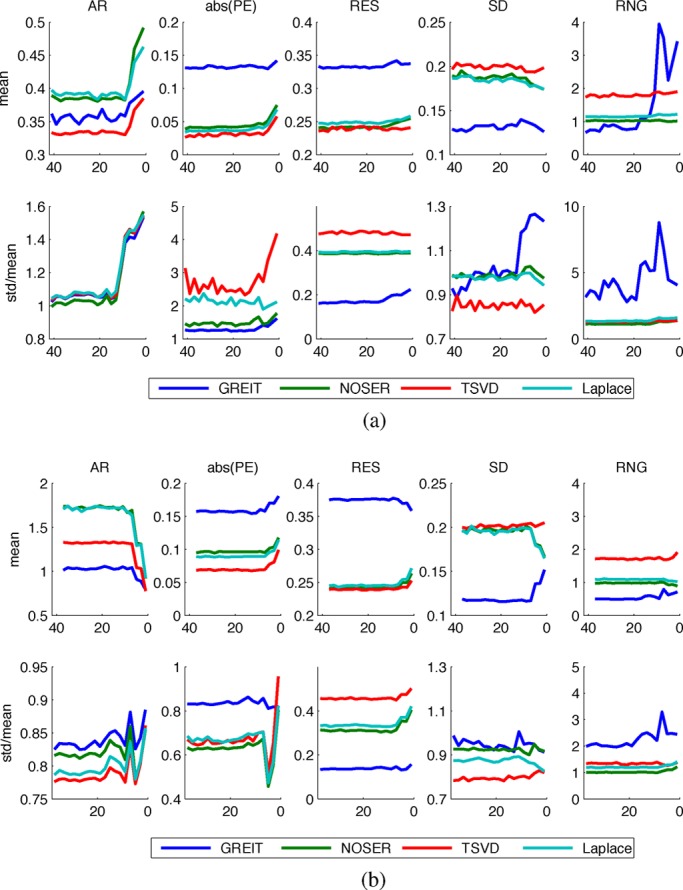
Dependence of algorithm performance measures on the number of Fourier coefficients used to describe the model shape. (a) Porcine thorax models. (b) Human thorax models.

exhibit the least artifacts but also the smoothest boundaries.

### B. Performance Measure Analysis

**Fig. 6.**
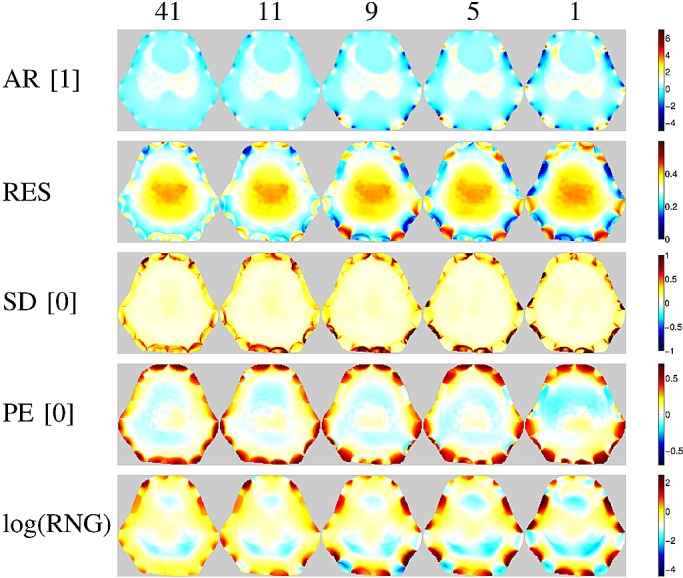
Figures of merit maps for the GREIT algorithm using five different porcine thorax model shapes (same as on Fig. 1). Numbers in brackets indicate the desired value of each parameter, represented as white in the color scale of the corresponding images.

Fig. 5 depicts for both models the mean and standard deviation (normalized to the mean) across the map of each performance measure as a function of the number of Fourier coefficients retained to describe the model shape, while sample performance measure maps of GREIT reconstructions on selected porcine thorax model shapes are presented in Fig. 6. With few exceptions, the performance measures visibly worsen as shapes become smoother, but not until the descriptor is truncated to below 13 coefficients for the porcine thorax $(\Delta S = 2.77\hbox{\%})$ and seven coefficients for the human chest shape $(\Delta S = 3.91\hbox{\%})$, cf. Fig. 2(d). This corresponds to approximately 4% difference in model shape $\Delta S$ (for the porcine shape described by 11 coefficient $\Delta S = 4.32\hbox{\%}$). None of the studied algorithms are immune to the effect.

**Fig. 7.**
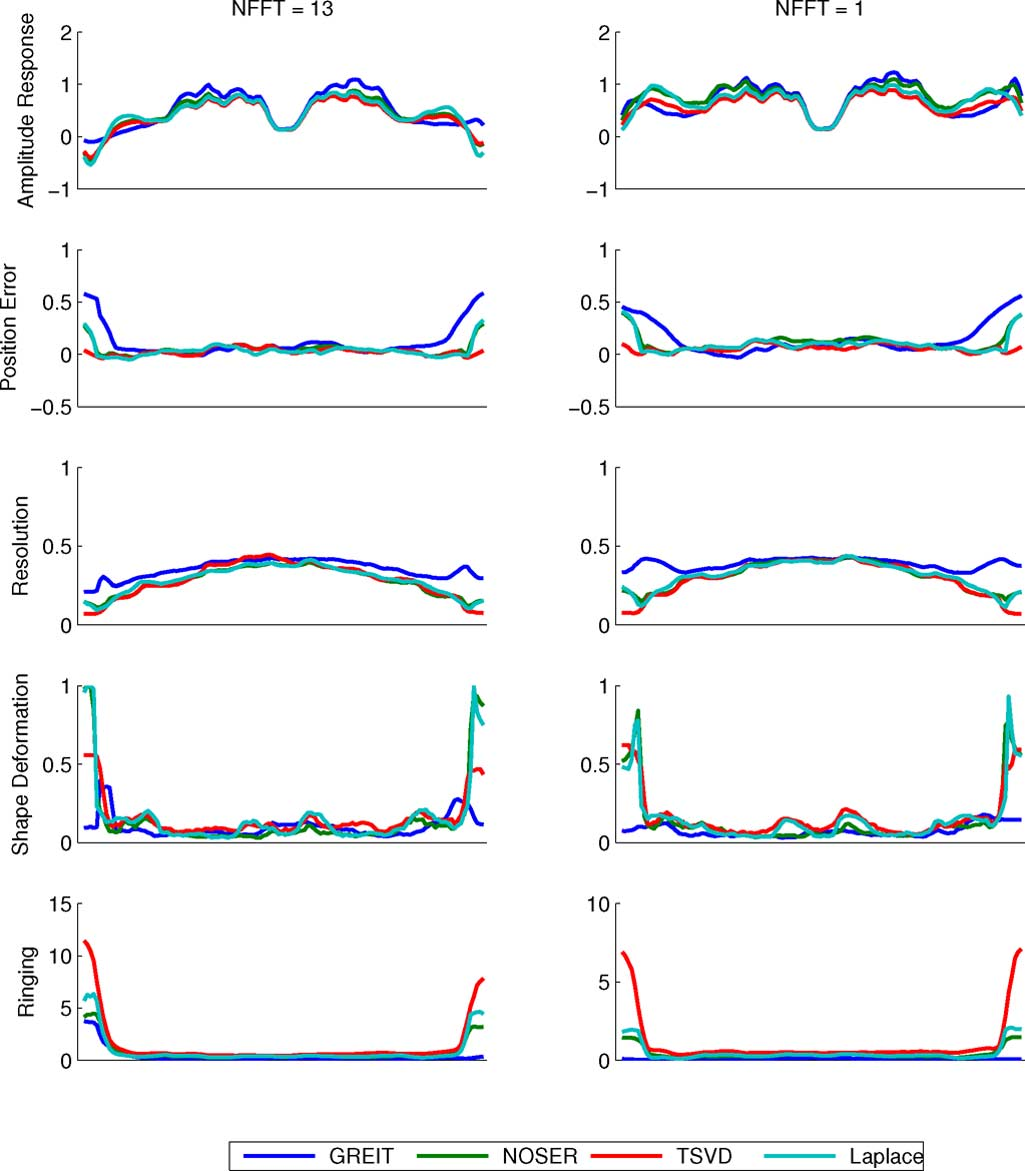
Horizontal cuts through the figure of merit maps for the porcine thorax models with shapes defined by (a) 13 and (b) 1 Fourier coefficient(s).

**Fig. 8.**
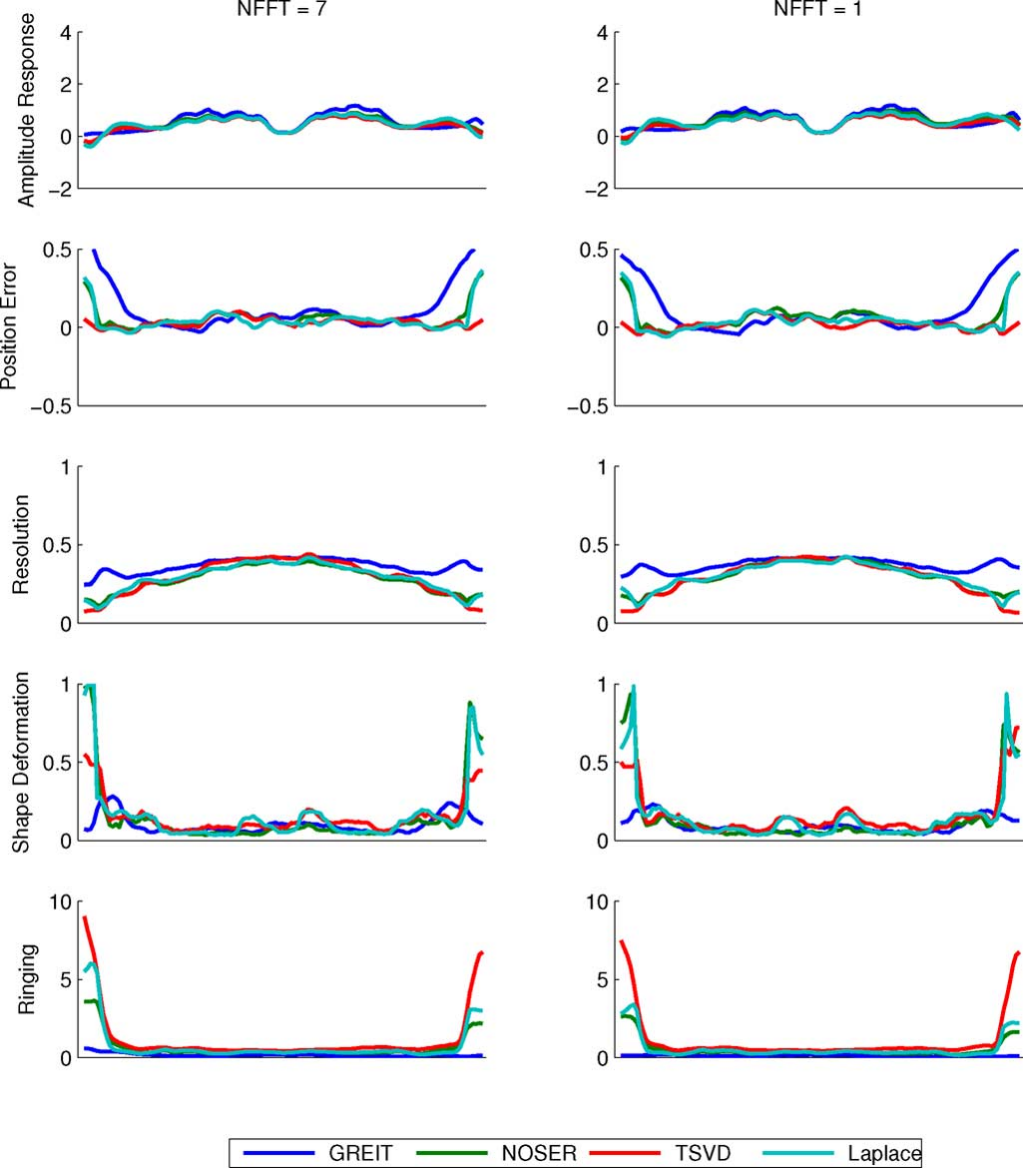
Horizontal cuts through the figure of merit maps for the human thorax models with shapes defined by (a) 7 and (b) 1 Fourier coefficient(s).

The performance of the two GN solvers is very similar. The GREIT algorithm stands out for its higher (i.e., worse) but more uniform resolution (cf. Figs. 7 and 8), lower ringing and shape deformation. It also has higher position error close to the boundary than other algorithms, which means that changes close to the boundary are reconstructed more centrally than they ought

to. The TSVD algorithm exhibits low average amplitude response, high shape deformation, high ringing as well as lowest and most variable resolution—low (i.e., good) at the boundary and high in the center.

## IV. Discussion

### A. Figures of Merit

In [10] a group of domain experts defined the desired characteristics of an EIT reconstruction algorithm as (in order of importance): 1) uniform amplitude response (AR), 2) small and uniform position error (PE), 3) low and uniform ringing (RNG), 4) uniform resolution (RES), 5) small shape deformation (SD), and 6) small RES. These are discussed below in turn.

The behavior of amplitude response is similar for all tested algorithms. In general, AR was not uniform across the image. Higher values were recorded within the lung as compared with the surrounding tissue, as shown on Figs. 1, 7 and 8. The results suggest that conductivity changes in less conductive tissues (e.g., inflated lung) are overestimated. This effect has potentially far reaching consequences for ventilation monitoring in patients with atelectasis (lung collapse), where EIT could underestimate ventilation in the more conductive collapsed regions of the lung. We also observed that AR is not uniform close to the boundary—it is high in the vicinity of the electrodes and low between them. This could distort the lung shape in some cases and be one of the sources of boundary artifacts in EIT images.

In terms of position error, the GREIT algorithm seems to have the lowest ranking performance. Although in the center of the image its performance is on a par or exceeds those of the other algorithms, objects that are close to the boundary but between electrodes are reconstructed deeper in the body than they ought to be. Because in thorax imaging the signals of interest originate primarily deeper within the body, this should not present a clinically significant issue. The TSVD algorithm performs best on this figure of merit.

Ringing displays high sensitivity to shape mismatch, especially for the GREIT algorithm. While in general GREIT displays lower RNG than the other algorithms, RNG increases pronouncedly for $\Delta S$ values in excess of 4%, especially at the boundary between the electrodes. The TSVD algorithm, intuitively similar to a boxcar filter in signal processing, produces the most ringing.

A nonuniform resolution could lead to a distorted shape and incorrect position of a reconstructed target. Amongst the evaluated algorithms, GREIT exhibits the most uniform resolution for both models. As the shape mismatch increases, RES becomes less uniform, but the non-uniformities seem to be limited to the boundary (cf. Fig. 6), and thus have limited impact on the shape deformation for changes of clinical interest in the chest.

Partially as a consequence of its uniform resolution, GREIT also features the least shape deformation. Although inaccuracies close to the boundary are present in all algorithms, they are least pronounced in GREIT.

Small resolution, i.e., the ability to distinguish nearby targets, is the lowest priority figure of merit in the GREIT framework [10]. It is, therefore, not surprising that GREIT performs worst on this criterion. However, the GREIT framework allows for several parameters in the calculation of the reconstruction matrix. A systematic investigation into those parameters could yield optimal values that improve the resolution.

### B. Recommendations

Our results demonstrate that shape mismatch has a strong detrimental effect on the quality of EIT reconstructions, including effects that are not apparent as artifacts but nonetheless can influence the analysis of EIT images. Therefore, we recommend that in both clinical and research applications EIT data be reconstructed on models closely resembling the actual shape of the body and reflecting the true positions of the electrodes. Our observation that shape mismatch of up to 4% has little impact on the quality of the reconstructions means that the shape does not need to match exactly and hence calculations may be simplified by smoothing over the finer detail.

### C. Clinical Practice

In clinical practice, the true shape of the thorax would be best represented by a cross-sectional image obtained by computed tomography (CT) or magnetic resonance imaging (MRI). Some patients may have prior imaging of the lungs using CT or MRI. Even if no prior pulmonary imaging was obtained, often abdominal CT scans contain a few images of the caudal part of the lung and could be utilized. If no prior imaging is available, the physician could choose the appropriate shape of the thorax from a set of predefined shapes based on measurements of a patient's height, weight, BMI, chest circumference or other easy to measure variables. Measurements like chest circumference, as well as 2-D measurements of the anterior–posterior and lateral dimension can be easily obtained at the bedside and can be helpful in determining the approximate shape. Calculating the patient's body mass index (BMI) can provide the investigator with reliable data on whether the patient is overweight (increased amount of soft tissue and body fat around the thorax), normal in weight or underweight (decreased amount of soft tissue and body fat around the thorax).

## V. Future Research

In the course of this study, we identified several issues that merit further investigation. First is the observed differential amplification by normalized difference EIT reconstruction algorithms of conductivity changes in the lungs and other tissues. Future research should seek to uncover the causes of this phenomenon, asses its impact on EIT images and devise strategies to reduce or correct for it. Second, for safety and practical reasons clinical use of EIT cannot depend on a prior examination with an anatomical modality used to build patient-specific models. Instead, efforts should be directed at developing other methods of measuring the patients' shape or identifying easy to measure physiological parameters that could be used to build an approximate FEM model or choose one from a library of ready-made models, and developing the required tools. The practicality of the latter approach, which we plan to explore in the near future, will depend largely on the required size of such a model library. The results of the present study are a first step towards estimating the requisite number of models, but further research into the variability of thorax shape and the number of factors that predict it is also needed.

Further to using the correct model shape, chest EIT should also account for breathing motion and associated with it changes in body shape and electrode positions, which have been shown to strongly influence the measured signal [8]. Future research should offer a characterization of these errors and investigate the efficacy of measures to compensate for them including, but not limited to, monitoring electrode movement with multiple cameras or recovering it from EIT data itself (e.g., [20]).

### A. Limitations

In the present study, we only investigated model shapes obtained by gradually smoothing the correct anatomical shape until a circle was obtained. This allowed us to easily control and quantify the amount of shape mismatch and offered insights into the performance loss caused by using the circular shape in studies on humans and swine. However, we can draw no conclusions about which particular features of a subject's shape are the most important to preserve. Thus, a shape mismatch below the 4% threshold found in our study should not be interpreted as a sufficient or necessary condition for obtaining quality reconstructions in EIT.

Our study is further limited in that we only analyzed normalized linear difference imaging algorithms. Because these algorithms only reconstruct relative changes in conductivity rather than its absolute value, they are less sensitive to uncertainties in initial conditions such as shape, electrode positions, their size and conductivity. Since changes in conductivity are of primary interest in ventilation monitoring, and due to the mentioned advantages, difference imaging is the most widespread approach to thoracic EIT.

## VI. Conclusion

We present a systematic study into the effect of reconstructing EIT data using models not reflecting the true shape of the investigated body. We demonstrated that using a circular model, as is frequently done, has a strong detrimental effect on a number of desired characteristics of difference EIT reconstruction algorithms, impeding analysis of the resultant images and possibly skewing conclusions. However, we found that small shape mismatch is well tolerated by all tested algorithms, allowing the use of approximate rather than exact model shapes. Future research and development effort should concentrate on developing the requisite knowledge and methods to allow easy choice of an appropriate model shape for individual patients at the bedside.
